# Comparison of device-based therapy options for heart failure with preserved ejection fraction: a simulation study

**DOI:** 10.1038/s41598-022-09637-4

**Published:** 2022-04-06

**Authors:** Marcus Granegger, Christoph Gross, David Siemer, Andreas Escher, Sigrid Sandner, Martin Schweiger, Günther Laufer, Daniel Zimpfer

**Affiliations:** 1 Department of Cardiac Surgery, Medical University of Vienna, Währingergürtel 18-20, 1090 Vienna, Austria; 2 Pediatric Cardiovascular Surgery, University Children’s Hospital Zurich, Zurich, Switzerland

**Keywords:** Biomedical engineering, Cardiac device therapy

## Abstract

Successful therapy of heart failure with preserved ejection fraction (HFpEF) remains a major unmet clinical need. Device-based treatment approaches include the interatrial shunt device (IASD), conventional assist devices pumping blood from the left ventricle (LV-VAD) or the left atrium (LA-VAD) towards the aorta, and a valveless pulsatile assist device with a single cannula operating in co-pulsation with the native heart (CoPulse). Hemodynamics of two HFpEF subgroups during rest and exercise condition were translated into a lumped parameter model of the cardiovascular system. The numerical model was applied to assess the hemodynamic effect of each of the four device-based therapies. All four therapy options show a reduction in left atrial pressure during rest and exercise and in both subgroups (> 20%). IASDs concomitantly reduce cardiac output (CO) and shift the hemodynamic overload towards the pulmonary circulation. All three mechanical assist devices increase CO while reducing sympathetic activity. LV-VADs reduce end-systolic volume, indicating a high risk for suction events. The heterogeneity of the HFpEF population requires an individualized therapy approach based on the underlying hemodynamics. Whereas phenotypes with preserved CO may benefit most from an IASD device, HFpEF patients with reduced CO may be candidates for mechanical assist devices.

## Introduction

Around half of all heart failure (HF) patients suffer from heart failure with preserved ejection fraction (HFpEF), a condition of diminished cardiac function, albeit with normal left ventricular ejection fraction (LVEF)^1,2^. HFpEF presents as a multifactorial pathology with several contributing factors (e.g. hypertension, aging population, obesity) that are currently increasing in prevalence. Consequently, the prevalence of HFpEF is projected to steadily rise in the forthcoming decades^3,4^.

In HFpEF patients, exercise intolerance is recognized as a major determinant of reduced quality of life. In fact, many HFpEF patients are free of any deterioration in cardiopulmonary and/or vascular function at rest, with respective declines being unmasked during exercise^5,6^. Most HFpEF patients share the common feature of smaller left ventricular (LV) dimensions compared to those observed in patients suffering from heart failure with reduced ejection fraction (HFrEF). These small ventricular cavities are usually combined with impaired diastolic ventricular function^3^.

Despite differences in pathophysiology, clinical outcomes in HFpEF patients are similar to those reported for the HFrEF pathology^7^. However, to date, effective and safe treatment approaches for the heterogeneous HFpEF population remain a major unmet clinical need. On the one hand, the established effective pharmacological treatment has not yet proven as effective in HFpEF as in HFrEF. On the other hand, the success of device-based therapies in HFrEF could not be translated toward HFpEF pathologies. In severe HFpEF cases, cardiac transplantation would remain the only viable therapeutic option. However, multiple comorbidities in this elderly population may render cardiac transplantation prohibitive in many patients.

In recent years novel device-based therapeutic approaches have been under investigation for the treatment of the HFpEF population^8,9^. Interatrial shunt devices (IASDs) that are inserted via a percutaneous approach have been shown to effectively reduce left atrial pressure (LAP) in-silico^10^ and in-vivo^11,12^. However, to date, no prospective study has demonstrated a corresponding reduction in HF-related adverse events^13^. Spring like expanders implanted in the LV chamber assist early cardiac filling by storing elastic energy during systole and releasing it during diastole in terms of expansion. To date, clinical data on safety and efficacy of these devices, however, are not available^9^. Mechanical circulatory support (MCS) devices, predominantly in form of third generation continuous flow rotodynamic blood pumps (RBPs), have proven effective as a therapy to support HFrEF patients^14^, and may also be applicable to the HFpEF population. However, the particular anatomical settings in HFpEF patients presenting with small LV cavities have triggered concerns that continuous LV unloading with an RBP may cause suction events around the inflow cannula^15,16^. These concerns are reflected by the fact that only anecdotal clinical use of MCS in adult patients with small ventricular dimensions has been reported in literature^17^.

Moscato et al.^16^ evaluated the use of RBPs in classical LV apical configuration (LV-VAD) in HFpEF patients in a numerical study during rest and exercise. Burkhoff et al.^15^ suggested the use of partial circulatory support that decompresses the left atrium (LA) as an LA-VAD based on a computational analysis. Recently, the CoPulse system, a novel ventricular assist device operating in synchrony with the LV^18,19^, was introduced as a promising MCS option for HFpEF patients. All these studies were restricted to either the evaluation of resting conditions solely, a single HFpEF phenotype and/or a single support strategy.

The aim of this study was to comparatively assess the hemodynamic effect of four different device-based therapies (IASDs, LV-VAD, LA-VAD and the CoPulse) in two typical HFpEF phenotypes during rest and physical activity in a numerical lumped parameter model.

## Methods

The approach of this numerical study was to:

1. Accurately replicate hemodynamics during rest and physical activity of two different HFpEF phenotypes in a lumped parameter model of the cardiovascular system including an autoregulated exercise response.
2. To investigate the effect of four different device-based therapy approaches (IASDs, LV-VAD, LA-VAD and CoPulse) on the hemodynamics simulated by the same model at rest and during exercise.

### Numerical simulation

#### Cardiovascular model

A numerical model similar to the ones presented in previous studies^15,16,20,21^ was employed to simulate the cardiovascular system of typical HFpEF patients. All heart chambers were modeled having a time varying-elastance with nonlinear end-diastolic/end-systolic pressure–volume relationships (EDPVR/ESPVR).

The ESPVR was defined as a parabolic relation with the vertex at the coordinates (${V}_{sys}$, ${P}_{sys}$) and crossing the volume axis of the pressure–volume plane at a V_0_ of 0 ml^20^:

1 $$ESPVR=[1-\left(\frac{{V}_{sys}-V\left(t\right)}{{V}_{sys}- {V}_{0}}\right)]{\cdot P}_{sys}$$

where V_sys_ and V(t) denote the vertex volume coordinate of the nonlinear ESPVR^20^ and the instantaneous ventricular volume [ml], respectively, and P_sys_ denotes the ESPVR’s vertex pressure coordinate [mmHg].

For the EDPVR we used the empirically found equation as presented in Klotz et al.^22^:

2 $$EDP=\alpha \cdot {EDV}^{\beta }$$

with EDP and EDV denoting the end diastolic pressure and volume, respectively, and α and β being dimensionless parameters. Atrial EDPVRs were adjusted to reflect the typically enlarged volumes (EDV of 90 ml at LAP of 15 mmHg)^15^ of HFpEF patients. The ventricular elastance curve was adjusted to mimic the typically elevated time constant of 54 ms during the early relaxation phase observed in HFpEF patients^16^.

**Figure 1:**
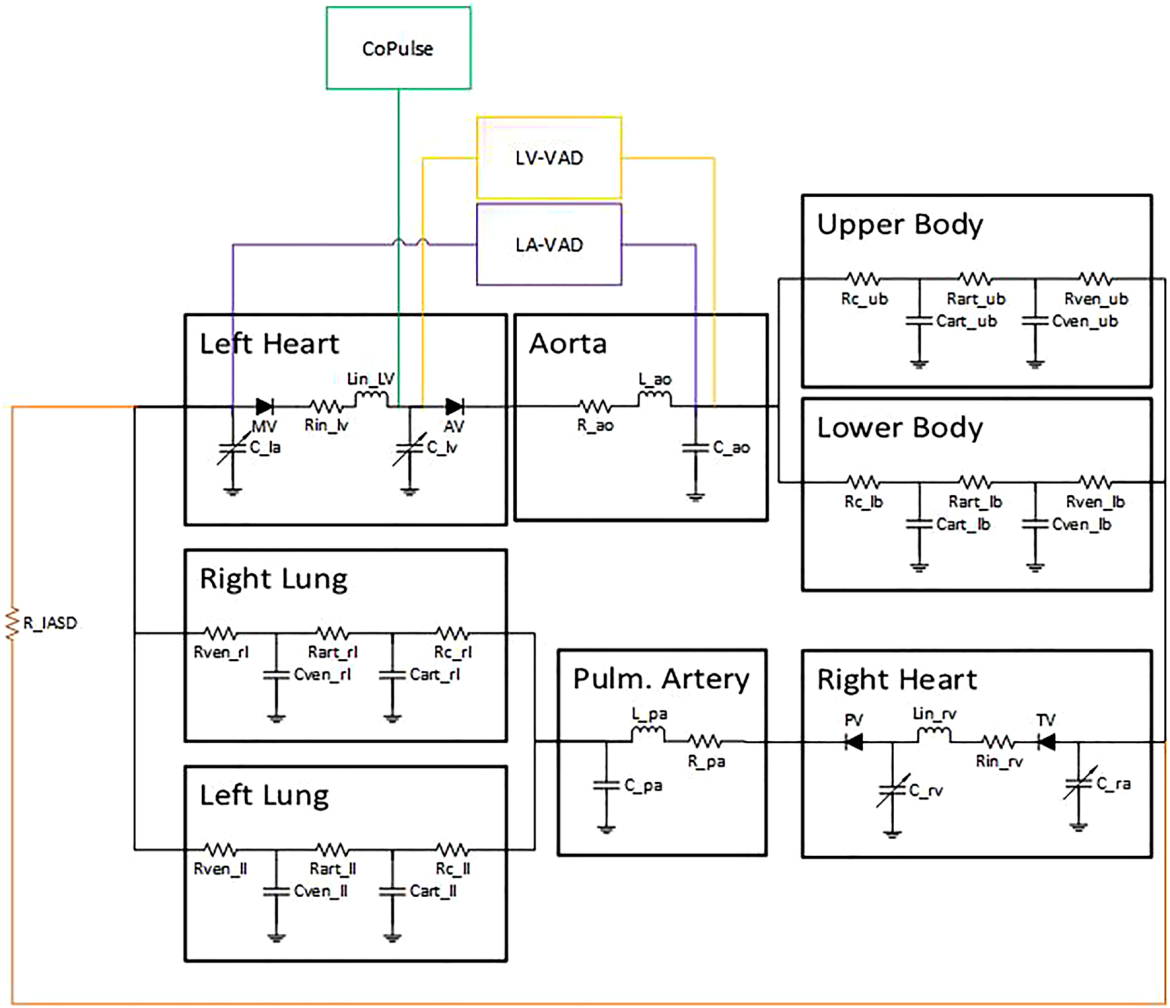
Electrical analogue of the numerical model of the cardiovascular system including the schematic pathways of the four different treatment strategies. Details on model parametrization are provided in Table 2.

To mimic the hydraulic properties of the cardiovascular system, the components of the arterial and venous system were modeled as 3- and 2-element Windkessel models^23^, respectively. In Fig. 1, an electrical analogue of the model is presented and indicates the model structure that was implemented in Matlab/Simulink (The MathWorks, Natick, MA, USA).

#### HFpEF phenotypes

In order to investigate the four device-based treatment options in typical HFpEF subgroups with different hemodynamic characteristics, we replicated hemodynamics of two distinct HFpEF phenotypes in the numerical model:

- Group 1: HFpEF patients with comparably low cardiac output (CO; 4.4 l/min) and elevated transpulmonary pressure gradient (TPG) of 10 mmHg (Group 1^24^) representative of the smaller subgroup with infiltrative and restrictive cardiomyopathies^25^.
- Group 2: HFpEF patients with elevated CO (6.3 l/min) and normal TPG of 4 mmHg (Group 2^16^) representative of the larger subgroup with one or more co-morbid conditions such as hypertension, obesity, diabetes and others^25^.

Resting and exercise hemodynamics were derived from Clemmensen et al.^24^ for Group 1 and from Moscato et al.^16^ for Group 2 and are depicted in Table 1. All model parameters were adjusted to match the literature data within the IQR or ± 1.5 SDs: In a first step, vascular resistances were derived from mean pressure and flow parameters in analogy to Ohm’s law. Second, the filling status in terms of mean circulatory filling pressure (MCFP) was adapted to achieve the required CO approximated by Guyton’s venous return theory and with the assumption of a venous resistance of 0.07 mmHg s/ml^18,20^. Third, we parametrized Eqs. (1) and (2) based on ventricular volumes and pressures provided in Table 1 and the assumption of a V_0_ of 0 mmHg. In a next step, vascular compliances were adapted to achieve the systolic and diastolic arterial pressures with the assumption of a total body compliance of 120 ml/mmHg^18,20,26^. Table 2 summarizes the parameters, which were eventually iteratively adapted to accurately replicate the hemodynamics of the two subgroups.

**Table 1:** Hemodynamics of Groups 1 and 2 at rest and exercise.

	Group 1		Group2	
	Rest			
	Literature^24^ n = 24	Simulation	Literature^16^ n = 264	Simulation
EDV (ml)	93 (73–108)	100.1	143 ± 20	142.8
ESV (ml)	39 (31–60)	41.5	55 ± 18	52.0
EF (%)	59 (45–63)	58.6	65 ± 12	63.6
MAP (mmHg)	91 ± 10	93.4	105 ± 14	108.0
HR (bpm)	76 (64–84)	76.5	71 ± 9	70.0
RAP (mmHg)	6 (5–8)	6.3	6 ± 2	6.6
PAP (mmHg)	25(18–29)	26.7	19 ± 3	22.0
PCWP/LAP (mmHg)	15 (11–20)	16.5	15 ± 7	16.7
CO (l/min)	4.4 ± 1.1	4.5	6.26 ± 0.85	6.34

	Exercise			
	Literature^24^ n = 24	Simulation	Literature^16^ n = 46	Simulation
EDV (ml)	–	114.5	158 ± 50	157.1
ESV (ml)	–	50.2	66 ± 45	63.3
EF (%)	–	56.2	58 ± 22	59.7
MAP (mmHg)	104 ± 28	108.5	123 ± 14	123.2
HR (bpm)	125 (105–141)	126.9	103 ± 14	102.6
RAP (mmHg)	14 (10–19)	17.3	14 ± 4	13.6
PAP (mmHg)	45 (40–52)	47.5	41 ± 6	32.9
PCWP/LAP (mmHg)	32 (25–35)	31.5	26 ± 4	25.0
CO (l/min)	7.7 ± 3.8	8.3	9.6 ± 2.0	9.70

*EDV* end diastolic volume, *ESV* end systolic volume, *EF* ejection fraction, *MAP* mean arterial pressure, *HR* heart rate, *RAP* right atrial pressure, *PAP* pulmonary arterial pressure, *PCWP* pulmonary capillary wedge pressure, *LAP* left atrial pressure, *CO* cardiac output.

**Table 2:** Values of parameters of the numerical model to achieve the typical resting hemodynamic condition.

Parameter	Description	Group 1	Group 2
R_ao	Resistance aorta ascend. (mmHg s/ml)	0.0075	0.0075
C_ao	Compliance aorta ascend. (ml/mmHg)	0.1619	
L_ao/L_pa	Inertance aorta ascend./pulm. artery (mmHg s^2^/ml)	5.5669 × 10^–5^	
Rc_ub/Rc_lb	Characteristic resistances upper/lower body (mmHg s/ml)	0.0384/0.0154	0.0308/0.0123
Cart_ub/Cart_lb	Arterial compliance upper/lower body (ml/mmHg)	0.1619/0.4857	
Rart_ub/Rart_lb	Arterial resistance upper/lower body (mmHg s/ml)	3.813/1.5205	3.0453/1.2181
Cven_ub/Cven_lb	Venous compliance upper/lower body (ml/mmHg)	25.2967/75.8929	
Rven_ub/Rven_lb	Venous resistance upper/lower body (mmHg s/ml)	0.245/0.098	0.245/0.098
R_pa	Resistance pulmonary artery (mmHg s/ml)	0.0075	
C_pa	Compliance pulmonary artery (ml/mmHg)	0.4909	
Rc_LL/Rc_RL	Characteristic resistances left/right lung (mmHg s/ml)	0.0026	0.0011
Cart_LL/Cart_RL	Arterial compliance left/right lung (ml/mmHg)	0.9818	
Rart_LL/Rart_RL	Arterial resistance left/right lung (mmHg s/ml)	0.2528	0.1059
Cven_LL/Cven_RL	Venous compliance left/right lung (ml/mmHg)	7.7727	
Rven_LL/Rven_RL	Venous resistance left/right lung (mmHg s/ml)	0.0192	0.0081
Lin_rv/Lin_lv	Ventricular inflow inertances (mmHg s^2^/ml)	2.78344 × 10^–5^	
Rin_rv/Rin_lv	Ventricular inflow resistances (mmHg s/ml)	0.001250	
MCFP	Mean circulatory filling pressure (mmHg)	12.7	14.3
LV V_sys	Vertex coordinate of the ESPVR (ml)	140	150
LV P_sys	Vertex coordinate of the ESPVR (mmHg)	220	235
LV α/β	Dimensionless parameters of EDPVR	5.5742 × 10^–6^/3.3186	9.1293 × 10^–7^/3.4085
RV V_sys	Vertex coordinate of the ESPVR (ml)	190	180
RV P_sys	Vertex coordinate of the ESPVR (mmHg)	60	95
LA and RA α/β	Dimensionless parameters of EDPVR	2.9417 × 10^–5^/2.9139	

*Q* flow rate in ml/s, *LV* left ventricle, *RV* right ventricle, *LA* left atrium, *RA* right atrium.

In addition, simplified autoregulatory closed-loop control mechanisms were implemented following Ursino et al.^27^ and Moscato et al.^16^: the difference between the baroreceptor set point, the required arterial pressure, and the measured mean arterial pressure (MAP) served as the input signal for the baroreflex control of heart rate (HR), systemic vascular resistance (SVR), and shift of unstressed to stressed venous volume to adapt the MCFP^16^. Parameters of the autoregulatory mechanisms were iteratively adapted to achieve typical hemodynamic changes observed in HFpEF patients during exercise^16,24^. The resulting SVR control changed arterial resistance by 1% per each mmHg difference between setpoint and MAP in both groups. Unstressed venous volume was adapted by 130 (Group 1) or 75 ml (Group 2) per mmHg difference between setpoint and MAP. HR was determined by the function

3 $$HR=\frac{60}{\left[{T}_{min}+{T}_{max}*\mathit{exp}\left(dP*{G}_{HR}\right)\right]/[1+exp\left(dP*{G}_{HR}\right)]}$$

with ${T}_{min}$ representing the minimum heart period (0.3 and 0.5 s for Group 1 and 2, respectively), ${T}_{max}$ the maximum heart period (1.35 and 1.3 s for Group 1 and 2, respectively) and ${G}_{HR}$ denoting the gain factor (0.0983 and 0.1075 for Group 1 and 2, respectively) for the difference between setpoint and MAP (dP).

Exercise was triggered by a drop in SVR by 45% and an increase of the desired systemic MAP by 32% (Group 1) and 30% (Group 2) determined by the baroreceptor setpoint.

#### Device models

**Figure 2:**
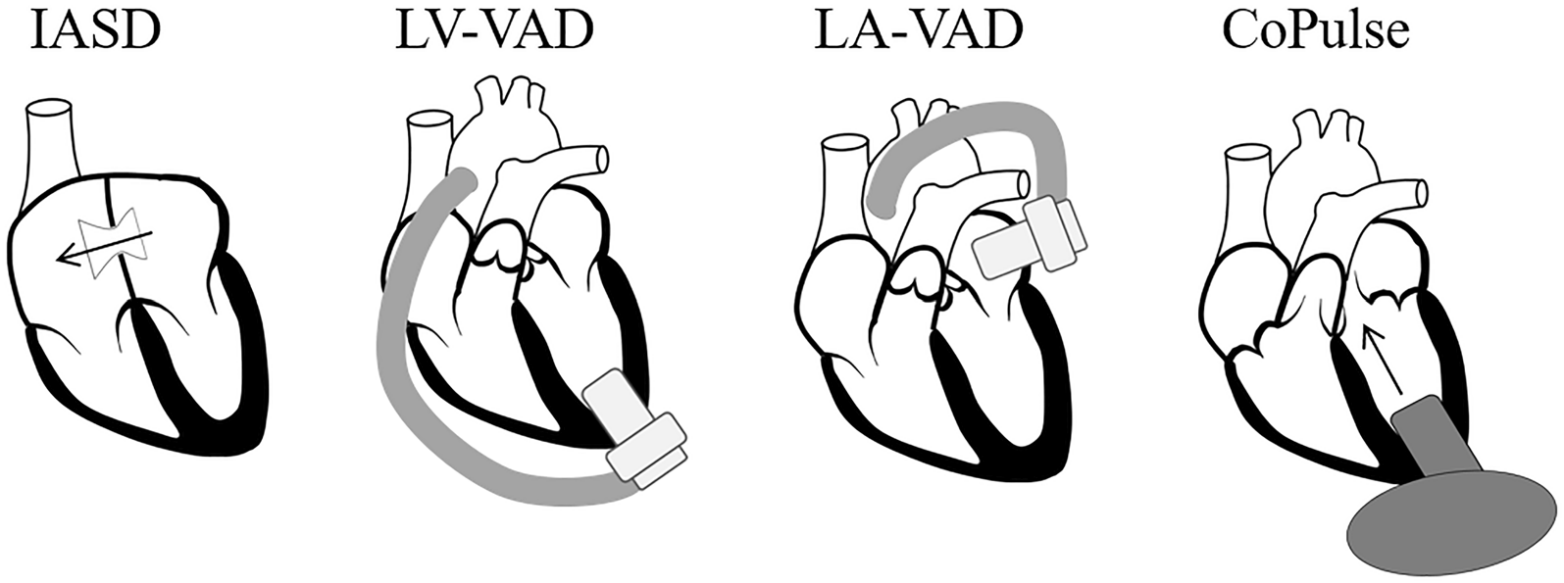
Schematic representation of the four investigated treatment strategies for HFpEF patients.

The numerical model of the cardiovascular system was applied to comparatively assess the hemodynamic effect of four different device-based treatment strategies with respect to corresponding unsupported (baseline) condition (Fig. 2).

- The *IASD* was modelled as a shunting resistance (0.1 mmHg s/ml*)* between the left and right atrium (Fig. 1) to permit decompression of the LA towards the pulmonary circulation^10^. This resistance value corresponds to a shunt diameter of approx. 8 mm which was previously shown to generate an adequate hemodynamic effect in HFpEF patients at rest and excercise^10^.
- The *HeartMate 3* was modelled as previously suggested^28^, accurately reflecting the static and dynamic properties of the device over the entire operating range. Two modes of support were examined with the pump inlet connected to the LV (*LV-VAD*) and the LA (*LA-VAD*), respectively (Fig. 1). In both modes, the pump outlet was connected to the aorta ascendens. Pump speed was adjusted to reduce the LAP below 10 mmHg and, at the same time, prevent backflow through the pump during diastole.
- The novel *CoPulse* system resembles a compliance chamber with a single, valveless cannula connected to the LV apex (Fig. 1). This device has a priming volume of 30 ml and pumps in co-pulsation with the native heart function, thereby supporting diastolic and systolic cardiac function in HFpEF patients^18,19^. The numerical model of the CoPulse device was validated in a previous study by comparing simulation with experimental results with marginal differences in hemodynamic findings (< 3.98%)^19^.

## Results

### HFpEF hemodynamics-baseline

Key hemodynamic parameters from literature in comparison to the simulated ones are provided in Table 1 for Group 1 and 2, respectively. All simulation results are within the IQR or 1.5*SD of literature data, respectively.

At rest, Group 1 features smaller EDVs (93 vs. 143 ml), less CO (4.4 vs. 6.3 l/min) and a 2.5-fold elevated TPG (10 vs. 4 mmHg) as compared to Group 2. During exercise, despite higher HR in Group 1 (125 vs. 103 bpm), CO remains lower (7.7 vs. 9.6 l/min) with elevated LAPs (32 vs. 26 mmHg) compared to Group 2.

### HFpEF hemodynamics with device-based therapy

**Figure 3:**
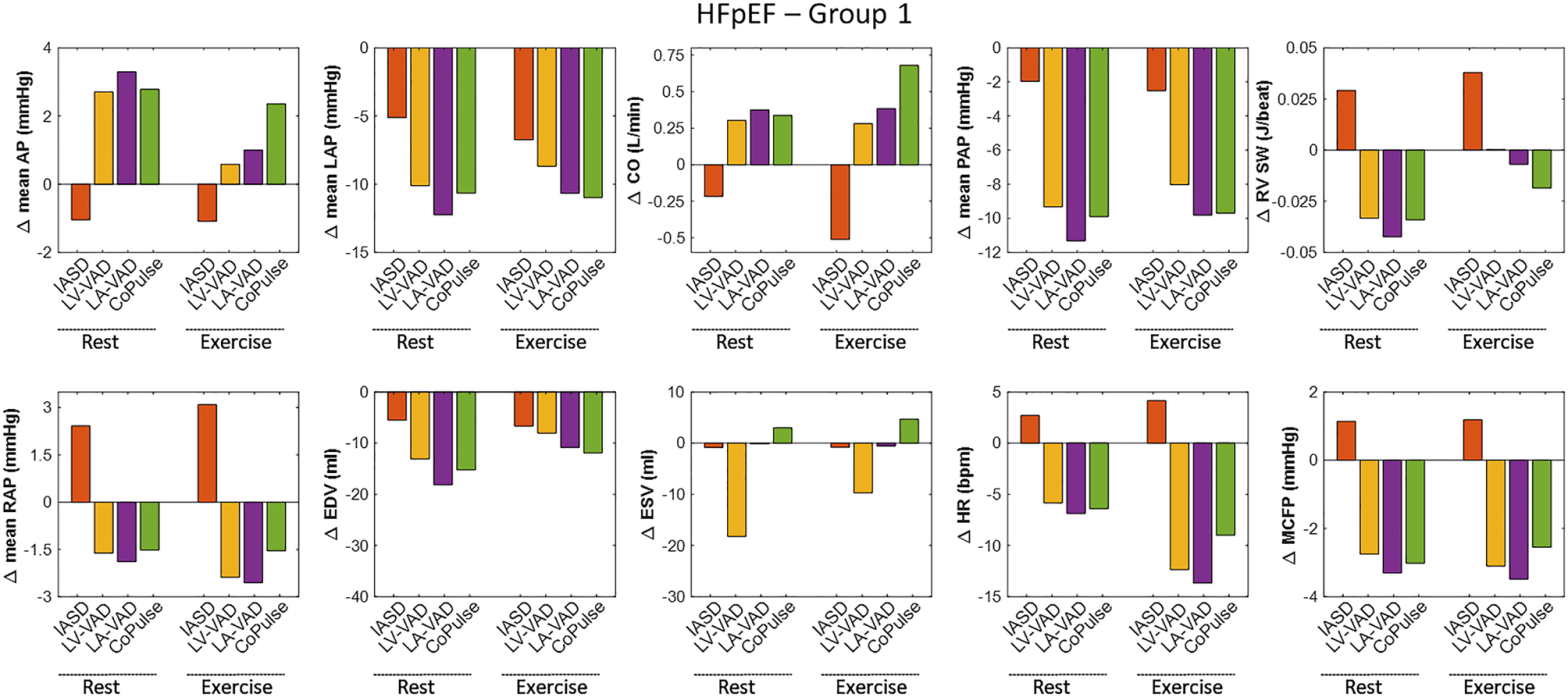
Hemodynamic effect of the four device-based treatment options in relation to baseline values of Group 1.

**Figure 4:**
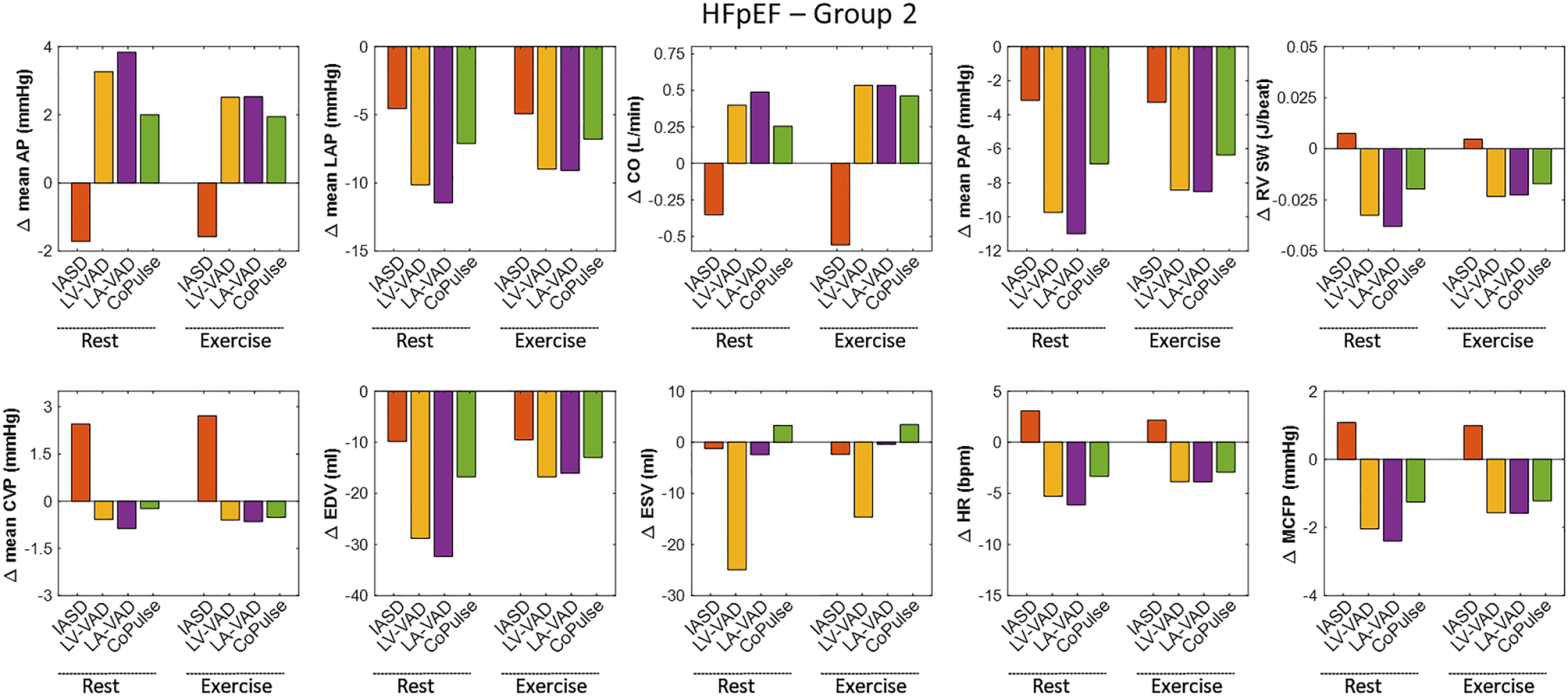
Hemodynamic effect of the four device-based treatment options in relation to baseline values of Group 2.

Simulated hemodynamics for both groups with the four device-based therapy options at rest and exercise are summarized in Figs. 3 and 4, respectively. Hemodynamic changes compared to baseline at rest and exercise showed the same trends with all devices in both groups (correlation coefficient > 0.9). Therefore, unless otherwise stated, results of rest and exercise conditions in terms of percentual changes are averaged across the two conditions (rest and exercise) as well as the two groups and presented as mean ± SD.

#### Systemic circulation

**Figure 5:**
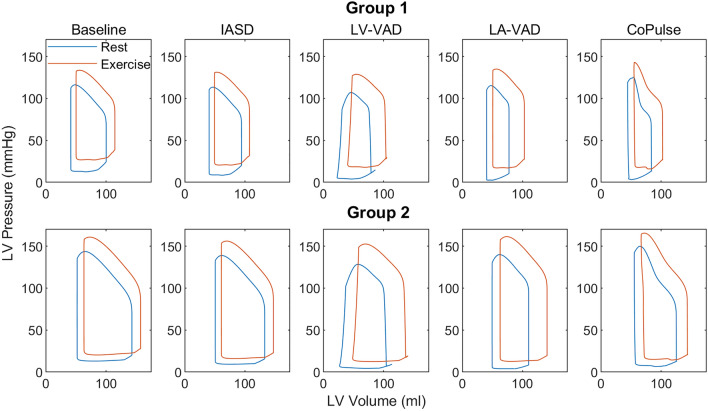
Pressure–volume loops at rest (blue) and exercise (red) for Group 1 (upper panels) and Group 2 (lower panels) visualizing the different working principles of the four different device-based treatment options and their effect on cardiac mechanics.

All four devices lead to LAP reduction of at least 20% (− 41.7 ± 18.0%). The three MCS strategies (LV-VAD, LA-VAD, CoPulse) promote CO (+ 6.1 ± 1.7%) with subsequent reduction in sympathetic activation, exemplified by lower HR (− 7.0 ± 2.6%). In contrast, with the IASD, CO is slightly reduced (− 5.6 ± 0.6%), and sympathetic activation elevated (HR: + 3.3 ± 0.9%). The four devices affect cardiac mechanics in a different way (see Fig. 5): End-systolic volume (ESV) is markedly reduced during LV-VAD support (− 33.6 ± 14.5%) and slightly elevated with the CoPulse system (+ 7.1 ± 1.7%), whereas LA-VAD and IASD do not affect ESV considerably (− 2.1 ± 1.5%).

With all devices, Group 1 indicates lower absolute ventricular volumes compared to Group 2 (ESV: 43.0 ± 9.5 vs. 52.7 ± 12.3 ml; EDV: 96.1 ± 10.4 vs. 132.1 ± 14.0 ml). Of note, although LAPs in the resting condition are comparable between both groups (7.0 ± 3.1 vs. 8.4 ± 3.1 mmHg), during exercise the LAP in Group 1 is considerably higher compared to Group 2 (22.2 ± 2.0 vs. 17.6 ± 2.0 mmHg).

#### Pulmonary circulation

All devices lead to a reduction of mean pulmonary artery pressure (PAP; − 24.8 ± 13.3%). Whereas for all MCS devices the drop in LAP is accompanied by a comparable decrease in PAP, with the IASD the decline in PAP is less pronounced compared to LAP changes (− 9.0 ± 3.7 vs. − 24.9 ± 5.2%). Further, all MCS devices cause a decline in right atrial pressure (RAP) and right ventricular stroke work (RVSW) (− 13.0 ± 9.2 and − 11.8 ± 7.1%, respectively). In contrast, with the IASD, RAP and RVSW are elevated by + 28.3 ± 10.9% and + 8.0 ± 5.6%, respectively.

With the IASD, Group 1 indicates a lower reduction of PAP (− 6.3 ± 1.5 vs. − 11.7 ± 3.1%) but more pronounced elevation of RVSW than Group 2 (+ 12.7 ± 1.9 vs. + 3.3 ± 1.5%).

#### Devices

The mean flow across the IASD from the left to the right atrium in Group 1 is 1.6 and 2.7 l/min for rest and exercise, respectively. In Group 2 the shunt flow is 1.8 and 2.3 l/min for the two respective conditions. Pump speed settings for the HeartMate 3 are lower in Group 1 compared to Group 2 with 5000 and 5300 rpm vs. 5700 and 5900 rpm for LV-VAD and LA-VAD, respectively. Accordingly, pump flow rates are lower in Group 1 (see Table 3). Despite the required high-speed settings to prevent backflow during diastole at rest (LAPs of approx. 5 mmHg), the LA-VAD setting in Group 2 lead to backflow during exercise with flow troughs of up to − 0.9 l/min.

**Table 3:** Pump settings and resulting flow rates for the two HFpEF groups at rest and exercise.

	Pump speed (rpm)	Pump flow-rest (l/min)	Pump flow-exercise (l/min)
Group 1—LV-VAD	5000	3.5	4.1
Group 1—LA-VAD	5300	2.1	2.5
Group 2—LV-VAD	5700	5.0	4.9
Group 2—LA-VAD	5900	3.0	2.5 (backflow)

## Discussion

The common objective of most device-based treatment approaches for HFpEF patients is to reduce LAP in order to normalize hemodynamics and thus relieve lung congestion and symptoms of dyspnea while preventing adverse pulmonary remodeling^15,29^. The use of traditional MCS approaches in form of RBP technology in HFpEF patients remains difficult as this pathophysiology requires minimal interference of the device with the typically small ventricular cavity in order to facilitate optimal operation and prevent suction events^15,16^.

Here, we comparatively assessed the hemodynamic effect of four device-based treatment strategies in an advanced numerical model: the IASD, LV-VAD, LA-VAD and the CoPulse system. All systems effectively reduced LAP during rest and exercise in both HFpEF groups. PAP decreased accordingly, however, with a lesser effect observed for the IASD compared to the three MCS devices (LV-VAD, LA-VAD, CoPulse). The different mechanisms of IASD and MCS devices were reflected by opposing trends in CO, sympathetic activity, as well as right ventricular pre- and workload.

Although the lack of standardized hemodynamic HFpEF phenotypes renders a quantitative comparison to other studies difficult, our findings with the IASD are consistent with previous simulation studies^10^ and clinical evidence^30,31^. The shunt flow across the IASD is in a similar range than simulated for a HFpEF population similar to Group 2 at rest and exercise (1.4 vs 1.8 l/min and 2.8 vs 2.3 l/min, respectively)^10^. In line with the trends observed in clinical studies with the IASD^30,31^, the LAP reduction comes at the cost of elevated right ventricular pre- and workload (RAP and RVSW, respectively). Our results show that the burden on the right heart is more pronounced in patients with higher pulmonary vascular resistance (PVR) and weaker right ventricular function (Group 2). This indicates that the IASD may be more effective in patients with lower PVRs and maintained right ventricular function. In addition, our approach to incorporate baroreflex function in the numerical model resembled clinically reported hemodynamic effects with the IASD^30,31^ (including a slight increase in HR (2–4 bpm) and a marginal change of − 2 mmHg in MAP) more closely than previous numerical models without baroreflex function (constant HR and more than − 10 mmHg drop in MAP)^10^. These improvements can be explained by the sympathetic stimulation of the autoregulatory baroreflex mechanisms as a response to a drop in systemic CO caused by the shunt flow across the IASD. Of note, the clinically observed trend towards even higher systemic COs and MAPs with the IASD in place could still not be mimicked by the numerical model. All these findings support the hypothesis that the IASD may be a viable treatment option for selected HFpEF patients with preserved CO, low PVR and good right ventricular function (e.g. Group 2).

All three MCS options resulted in lower LV preload (reduced LAP and EDV) and an increase in CO with subsequent reduction of sympathetic activity during rest and exercise in both groups. Consequently, RAP was slightly reduced, and arterial pressure elevated compared to baseline. These hemodynamic trends are consistent with the results of Moscato et al.^16^ investigating the use of an LV-VAD in Group 2 within a similar numerical model including a baroreflex mechanism. Further, the results of Burkhoff et al.^15^, comparing the use of LV- and LA-VAD in patient populations similar to Group 1 and 2 in a numerical model without baroreflex, are qualitatively consistent with our observation in terms of hemodynamic trends and PV-loop morphology. Our results confirm the findings of these previous studies that LV-VAD excessively unloads the LV cavity during systole (low ESV) with a high risk for suction events around the inflow cannula^32^, whereas the ESV is only slightly reduced or even elevated for the LA-VAD and the CoPulse, respectively. These results imply that LA-VAD and CoPulse may be viable treatment options for HFpEF patients with reduced CO and/or elevated PVR (e.g. Group 1) with almost equivalent efficacy in terms of hemodynamic changes. The CoPulse system with a stroke volume of 30 ml improves hemodynamics similar to the LA-VAD. Of note, this system operates in co-pulsation to the native LV and therefore relies on the chronotropic response of the patient. The dependency of more unloading with higher HRs explains the observation that Group 1 benefits more from this device in terms of LAP reduction during exercise as compared to Group 2 (126 vs. 106 bpm).

Our results suggest that IASD, LA-VAD and CoPulse may be viable device-based treatment options to support selected HFpEF patients, while a small ventricular cavity may impair the efficacy of LV-VADs. The IASD, LA-VAD and CoPulse may serve different patient populations. Whereas the IASD may be most beneficial for patients with higher CO, lower PVR and a good right ventricular function, the two MCS strategies may be preferable in HFpEF patients with lower CO.

In this context, the consideration of an LA-VAD as potential RBP-based treatment option in selected HFpEF patients requires particular attention regarding pump speed setting and respective unloading. On the one hand, the objective to achieve an adequate unloading without negative pump flows during diastole led to low LAPs of < 7 mmHg at rest. On the other hand, at exercise, higher pump speeds would have been required for adequate unloading, especially in Group 1 with LAPs > 20 mmHg. Combined with the observation of backflow through the pump during diastole in Group 2 with LA-VAD treatment (see Table 3), this supports the need for specifically adapted RBPs for LA-VAD applications in HFpEF patients. Such devices may be optimized for low flow operation, featuring a comparably steep HQ curve to prevent backflow during diastole and a closed-loop physiologic control algorithm to adapt the pump to the patient’s need.

Whereas the IASD is commercially available and under clinical evaluation, the CoPulse system and dedicated devices optimized for LA-VAD application are currently not available. LA-VADs based on the well-known rotodynamic pumping principle are under development and may present a valuable treatment option for HFpEF patients in the near future. The novel CoPulse system, showing equivalent hemodynamic efficacy with LA-VAD in this simulation study, has yet to prove its durability and potential advantages in terms of hemocompatibility in future in-vivo studies.

In this study only two groups of the heterogenous HFpEF population with distinct hemodynamic characteristics were considered. However, the wide range of combinations of hemodynamic characteristics in HFpEF patients (right heart function, pulmonary or systemic hypertension, volume overload, and others) renders a personalized selection of device-based treatment option indispensable. Further research should focus on hemodynamic phenomapping and definition of adequate hemodynamics to select the appropriate and individualized treatment strategy.

### Limitations

This simulation study has intrinsic limitations of numerical modelling approaches: whereas most parameters of the basic numerical model were computed based on the available data from literature, the simplified baroreflex function was only empirically tuned to match the hemodynamics at rest and exercise. Therefore, the model does not capture exercise and baroreflex mechanisms in its entire complexity but focuses on the resemblance of realistic trends in hemodynamic parameters only. Of note, imperfection of available clinical data makes assumptions and manual imputation of many parameters unavoidable.

Long-term auto-regulation effects such as the renin-angiotensin system are not considered in this model. Nevertheless, such models proved valuable to understand mechanisms and effects related to device-based therapy in HF patients.

In this study we did not consider LV expanders as their functional principle cannot be accurately assessed by a lumped parameter model. More complex models combining lumped parameter modelling in combination with Finite Element Modeling^33^ may provide insights into the hemodynamic effect of these devices.

Another important therapy objective for HF treatment is the ability to stop or even reverse adverse remodelling of the cardiovascular system as a response to hemodynamic stimuli. To date it remains unclear how any of these devices affect the heart in terms of structural and functional remodelling.
